# Reduction of Dislocation of the Femur by Manipulation

**Published:** 1858-04

**Authors:** Frank H. Hamilton

**Affiliations:** Buffalo, N. Y.


ART. III.—*Dislocation of the Femur upon the Dorsum Ilii. Reduction by Manipulation.* By Frank H. Hamilton, M. D., Buffalo, N. Y.

John Caswell, æt. 28, was at work, Jan. 7, 1858, at the bottom of a well; he was bent forwards, with his body at a right angle with his legs, when a large bucket, holding 500 weight of earth, fell upon his back and hips.

He was carried home and a physician was called, who, discovering the nature of his injury, declined to interfere, but directed him to the hospital. For some reason, not fully explained, he was not brought to the hospital until the 13th of January, six days after the accident. Until this time no attempt had been made to reduce the dislocation.

I found him a short, muscular man, with his left thigh dislocated upon the dorsum ilii. The whole limb was considerably swollen.

In presence of the class of medical students, and after having placed him moderately under the influence of chloroform, we proceeded to attempt the reduction.

I first flexed the leg upon the thigh, and then carried the knee towards the body, the thigh remaining in a state of adduction. When the flexion of the thigh upon the body was complete, I abducted and rotated the thigh outwards moderately, and then gradually brought both thigh and leg down to the table upon which he was lying.

The result showed that the effort was unsuccessful. The dislocation still continued as before.

I immediately proceeded to repeat the same manipulation, and with the same result, except that when the thigh began to descend, a sudden click indicated that the head of the femur had slipped off from the margin of the acetabulum, but that it had not fallen into it.

The chloroform was now suspended and the manipulations were repeated three times more, with only slight variations; but the reduction was not accomplished.

On the sixth trial the chloroform was resumed, and Dr. Cushing, who was present, made the manipulation. Instead of simply pressing the limb in various directions, at my instance, he stood over the patient and lifted or pulled upon the thigh at the same time that he flexed and abducted the limb. When the leg was brought down to the table the head of the femur was found to rest upon the foramen thyroideum; the thigh being forcibly abducted and rotated outwards.

Reversing the order of the movements I was able easily to restore the bone to its original position.

The seventh trial was made by myself in the same manner, with the preceding, except that when I supposed the head of the bone to be opposite the lower margin of the socket I did not permit the limb to turn either outwards or inwards, but while lifting at the knee with sufficient power to raise his hips from the table, I brought the leg down straight until the extension was complete. I did not feel any slipping or sensation of any kind as the limb was moved, but when the manœuvre was completed we found the bone had resumed its place.

We laid the patient in bed, upon his back, with his knees tied tightly together. No pain or inflammation of consequence followed. In two weeks he left the hospital. Whether he was then able to walk or not, I cannot say, but I think it very probable that he was.

